# Levels of Growth Differentiation Factor 15 Correlated with Metabolic Dysfunction-Associated Steatotic Liver Disease in Children

**DOI:** 10.3390/ijms26136486

**Published:** 2025-07-05

**Authors:** Antonella Mosca, Maria Rita Braghini, Giulia Andolina, Cristiano De Stefanis, Lucia Cesarini, Anna Pastore, Donatella Comparcola, Lidia Monti, Paola Francalanci, Clara Balsano, Andrea Pietrobattista, Anna Alisi, Nadia Panera

**Affiliations:** 1 Hepatology and Liver Transplant Unit, Bambino Gesù Children’s Hospital, IRCCS, 00165 Rome, Italy; antonella.mosca@opbg.net (A.M.); donatella.comparcola@opbg.net (D.C.); andrea.pietrobattista@opbg.net (A.P.); 2 Research Unit of Genetics of Complex Phenotypes, Bambino Gesù Children’s Hospital, IRCCS, 00165 Rome, Italy; mariarita.braghini@opbg.net (M.R.B.); giulia.andolina@opbg.net (G.A.); lucia.cesarini@opbg.net (L.C.); anna.pastore@opbg.net (A.P.); nadia.panera@opbg.net (N.P.); 3 Core Facilities, Bambino Gesù Children’s Hospital, IRCCS, 00165 Rome, Italy; cristiano.destefanis@opbg.net; 4 Diagnostic and Interventional Radiology Unit, Bambino Gesù Children’s Hospital, IRCCS, 00165 Rome, Italy; lidia.monti@opbg.net; 5 Molecular Pathology Research Unit, Bambino Gesù Children’s Hospital, IRCCS, 00165 Rome, Italy; paola.francalanci@opbg.net; 6 Geriatric Unit, Department of Life, Health and Environmental Sciences-MESVA, School of Emergency and Urgency Medicine, University of L’Aquila, 67100 L’Aquila, Italy; clara.balsano@univaq.it; 7 Francesco Balsano Foundation, Via Giovanni Battista Martini 6, 00198 Rome, Italy

**Keywords:** children, obesity, metabolic syndrome, insulin resistance, GDF15, hepatic steatosis, liver fibrosis, tissue injury, MASLD, MASH

## Abstract

Metabolic dysfunction-associated steatotic liver disease (MASLD) is the most common chronic progressive hepatopathy in children, and the identification of non-invasive biomarkers is urgently needed. Growth differentiation factor 15 (GDF15) was associated with MASLD in adults. In this study, we investigated the circulating and hepatic levels of GDF15 and their association with liver damage in pediatric MASLD and in a murine model. This observational study included 158 children with biopsy-proven MASLD. Patients with MASLD were categorized into two groups based on steatohepatitis (MASH) presence and evaluated for GDF15 circulating levels, while GDF15 hepatic levels were assessed only in a subset of patients. Children with MASLD exhibited higher levels of circulating GDF15 compared to the controls. Moreover, the MASH subgroup had significantly higher values of GDF15 compared to the Not-MASH subgroup. The GDF15 levels in the MASH subgroup showed a positive correlation with fibrosis. Finally, the hepatic expression of the GDF15 gene correlated with GDF15 circulating levels and with the hepatic expression of the COL1A1 and COL3A1 genes in 15 children with MASLD. In conclusion, our study demonstrated that GDF15 levels are associated with liver damage, reinforcing the potential role of GDF15 as a biomarker for MASLD-related fibrosis in children.

## 1. Introduction

Metabolic dysfunction-associated steatotic liver disease (MASLD) includes several subcategories of liver conditions ranging from simple steatosis to the more severe form of steatohepatitis (metabolic dysfunction-associated steatohepatitis, MASH), which may be associated with inflammation, hepatocyte ballooning, and fibrosis [1]. A recent analysis revealed a concerning rise in MASLD global prevalence among children, adolescents, and young adults, with no signs of slowing, thus becoming a worrying phenomenon in adults and youth. Indeed, it has been calculated that the prevalence of MASLD in children worldwide increased from 4.62% to 9.02% between 2000 and 2017 and is estimated to reach 30.7% by 2040 [2]. Moreover, MASLD also emerged as one of the most common causes of liver transplants in young adults [3].

In this scenario, the challenge lies in identifying early non-invasive biomarkers for MASLD diagnosis and prognosis, particularly for the identification of patients with MASH and stratification according to fibrosis severity. Multiple circulating molecules have been explored as biomarkers for liver injury in MASLD, but most of these results are generally less accurate than liver biopsy in detecting MASH and fibrosis, necessitating further research [4].

Recently, among the plethora of identified molecules, growth differentiation factor 15 (GDF15) has been explored in several pre-clinical and clinical studies as a potential biomarker for MASLD [5].

GDF15, a divergent member of the transforming growth factor β family, has been found to be involved in the control of several physiological processes, including appetite regulation, metabolism, cell and tissue survival, and immune homeostasis [6]. It is generally expressed at low levels in various tissues, including the liver, but its local and systemic expression significantly increases in pathological states, including obesity, tissue injury, inflammation, and malignancies [7]. Nowadays, it is emerging that GDF15 significantly influences the development and progression of MASLD by improving insulin resistance and attenuating hepatic steatosis, inflammation, and fibrosis [8]. A recent study by Girona et al. [9] found that higher levels of circulating GDF15 are associated with components of cardiometabolic risk in patients with metabolic conditions, including obesity, metabolic syndrome, diabetes, or cardiovascular disease. The serum GDF15 levels positively correlated with pathological features of MASLD, an atherogenic lipid profile, and an increased long-term risk of cardiovascular disease.

A study in a biopsy-proven MASLD cohort of adult subjects revealed that the circulating levels of GDF15 positively correlated with the severity of lobular inflammation, ballooning, and fibrosis, suggesting the potential of GDF15 as a biomarker of advanced fibrosis in MASLD [10]. In addition, a cross-sectional study in children and adolescents showed that the plasma levels of GDF15 were associated with intrahepatic fat content and plasma ALT (alanine aminotransferase) concentrations in overweight/obese patients with MASLD [11].

Hence, the purpose of our study was to investigate the changes in the circulating GDF15 levels in a pediatric cohort of children with a liver biopsy-proven diagnosis of MASLD and to evaluate the possible association of GDF15 with the different metabolic and hepatic features that characterize the disease. Moreover, we also explored the hepatic expression of the GDF15 gene in a subset of our pediatric cohort with MASH, in a liver dataset from adults with MASLD, and finally, in a diet-induced mouse model of MASH.

## 2. Results

### 2.1. GDF15 Plasma Levels Increase in Children with MASLD

**Figure 1:**
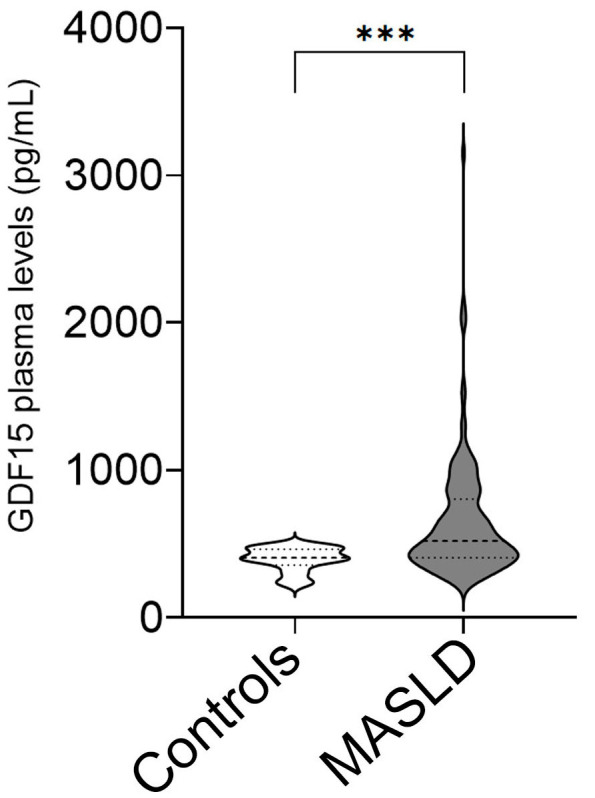
Plasma levels of GDF15 in the study population. The violin plot shows the comparison of the plasma GDF15 levels between 24 healthy subjects (controls) and 158 patients with MASLD (MASLD). Mann–Whitney U-test, *** *p* < 0.001.

This study involved 158 children and adolescents affected by MASLD, with a median age of 13 years, and 24 healthy control adolescents, with a median age of 12 years. The general characteristics of the study population are summarized in Table S2. Overall, patients with MASLD had significantly increased BMI (*p* < 0.0001) and levels of ALT (*p* < 0.017), AST (*p* = 0.034), TC (*p* = 0.032), LDL-C (*p* = 0.019), and triglycerides (*p* = 0.011), compared with individuals in the control group. Otherwise, there were no significant differences in age, sex, fasting glucose, and HDL-C between the two groups. Moreover, as shown in Figure 1, we found that the median circulating levels of GDF15 were 395.6 pg/mL in the control group and 640.0 pg/mL in the MASLD group, with a statistically significant difference between the groups (*p* < 0.0001).

As reported in Table S3, the evaluation of the correlation between GDF15 circulating levels and metabolic, anthropometric, and biochemical parameters in the pediatric MASLD group revealed a positive correlation with BMI, the levels of triglycerides, ALT, GGT, fasting insulin, and HOMA-IR, fibrosis, steatosis, and inflammation.

### 2.2. GDF15 Plasma Levels Are Associated with a More Severe Pattern of MASLD in Children

Histological data (Table S4) were used to divide the MASLD group into two subgroups, including patients without MASH (Not-MASH; n = 60, 28%) and patients with MASH (MASH, n = 98, 72%). When clinical characteristics and biochemical variables were compared between the two identified groups, it was found that the patients with MASH had significantly higher values of ALT, AST, LDL-C, fasting insulin, and HOMA-IR (*p* < 0.05) than the Not-MASH subgroup (Table S5).

**Figure 2:**
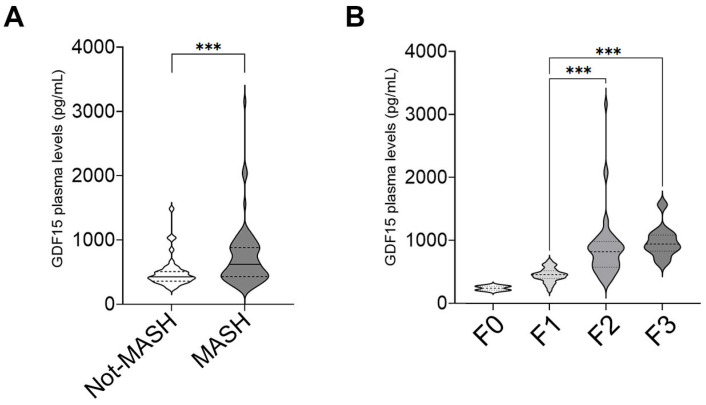
Plasma levels of GDF15 in MASLD patients according to MASH diagnosis. (**A**) The violin plot shows the comparison of the plasma GDF15 levels between patients without (Not-MASH) and with a MASH diagnosis (MASH). Mann–Whitney U-test, *** *p* < 0.001. (**B**) The violin plot shows the comparison of the plasma GDF15 levels between different stages of fibrosis in patients with MASH.

Moreover, the measurement of GDF15 plasma levels according to the MASH diagnosis showed that the median value was 426.2 pg/mL in the Not-MASH subgroup and increased significantly (*p* = 0.0004) to a median value of 628.1 pg/mL in the MASH group (Figure 2A).

We then performed a correlation study to evaluate whether GDF15 plasma levels were associated with metabolic parameters and liver damage in MASH. As shown in Table 1, GDF15 plasma levels were significantly correlated only with the levels of triglycerides (r = 0.22, *p* = 0.03). The correlation study with histological features showed that in patients with MASH, the plasma levels of GDF15 exhibited no correlation with steatosis, ballooning, and inflammation, while they were positively correlated with fibrosis (r = 0.48, *p* = 0.0001), with a significant increase in fibrosis stage 3 (Figure 2B).

At multivariate regression, adjusted for BMI, WC, and gender, GDF15 levels remained independently associated with triglyceride levels (β = 1.61, SE 2.5, *p* = 0.0129) and fibrosis (β = 9.2, SE 5.9, *p* = 0.0001). In addition, the independent association of GDF15 levels with the levels of fasting insulin (β = 2.03, SE = 1.67, *p* = 0.0042) and HOMA-IR values (β = 9.22, SE 2.22, *p* = 0.027) also emerged.

### 2.3. The Circulating Levels of GDF15 Correlate with Its Hepatic Levels in Children with MASLD

**Figure 3:**
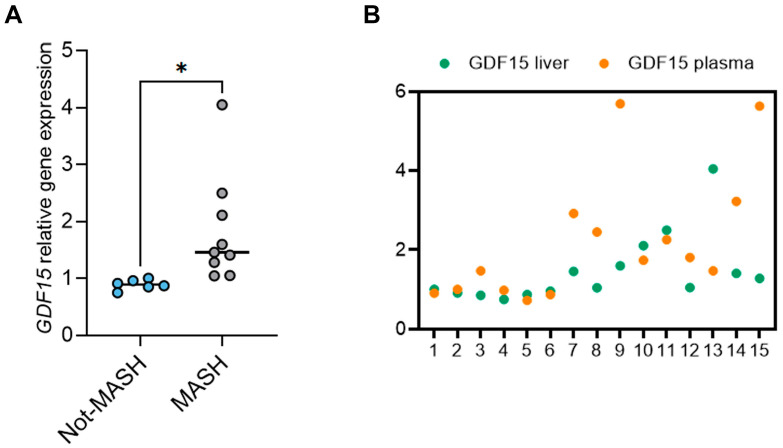
Plasma and hepatic expression of GDF15 in a subgroup of children with MASLD. (**A**) The graph shows the relative hepatic gene expression of GDF15 in 9 children with MASH diagnosis (MASH) vs. 6 children without MASH (Not-MASH), normalized to β-actin transcript expression. Unpaired t-test with Welch’s correction, * *p* < 0.05. (**B**) Scatter plot representation of the plasma and corresponding hepatic GDF15 levels in 9 MASH and 6 Not-MASH children.

Since it has been shown that, under experimental pathological conditions such as obesity, the liver, particularly the hepatocytes, contributes more than other organs to increasing the plasma levels of GDF15 [12], we further evaluated whether the changes in GDF15 plasma levels reflected its altered expression at the hepatic level in our patients. For this purpose, liver sections from a subset of 15 patients with MASLD were used for the analysis of GDF15 gene expression levels, which resulted to be higher in MASH (n = 9) than in Not-MASH (n = 6) livers (Figure 3A). In addition, as shown in Figure 3B, we found a significant positive correlation between the changes in the plasma and hepatic GDF15 levels (Spearman’s r = 0.61; *p* = 0.016).

### 2.4. The Hepatic Levels of GDF15 Transcripts Positively Correlate with the Levels of Fibrotic Genes in Humans with MASLD and in a Murine MASLD Model

**Figure 4:**
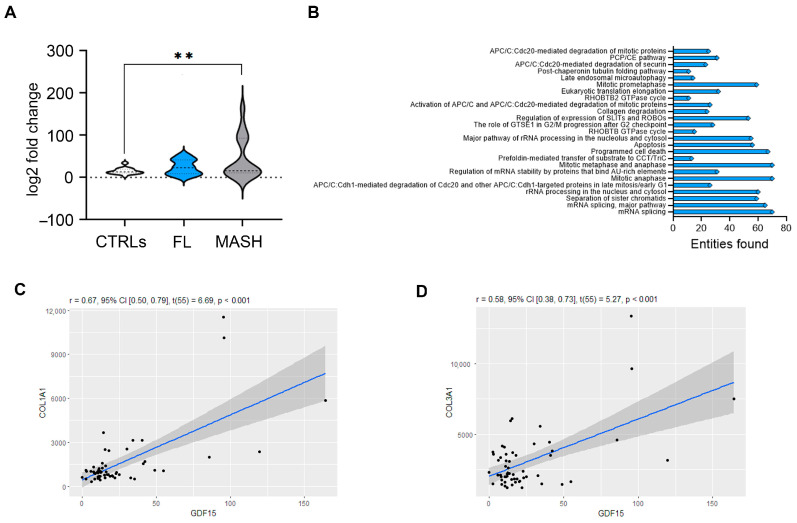
Hepatic expression of GDF15 and correlated genes in a gene expression dataset from adult patients. (**A**) Violin plot shows the fold-change (log2) hepatic expression of the GDF15 transcript in control subjects (CTRLs) and in patients with simple fatty liver (FL) or MASH (MASH) by using data retrieved from the GSE126848 dataset. One-way Anova with Bonferroni correction ** *p* < 0.001 (**B**) Pathways enrichment analysis by Reactome for genes significantly correlated with GDF15. Correlation analysis between the hepatic gene expression of GDF15 and that of (**C**) COL1A1 and (**D**) COL3A1 in the GSE126848 dataset.

Next, we performed a retrospective analysis of the GSE126848 adult RNA sequencing dataset (http://www.ncbi.nlm.nih.gov/geo/, accessed on 1 July 2024), which includes 26 healthy subjects (CTRLs), 15 patients with fatty liver (FL), and 16 with MASH. As shown in Figure 4A, the GDF15 transcripts were significantly upregulated in the liver of adult patients with MASH compared to that of CTRLs (*p* = 0.005). Interestingly, GDF15 expression was significantly positively correlated with the expression of 2791 genes (Table S6). A Reactome analysis (https://reactome.org, accessed on 3 July 2024) revealed that these identified genes belong to several pathways, of which the 25 most representative are shown in Figure 4B. Interestingly, among these pathways, the one involved in collagen degradation emerged, which includes the COL1A1 and COL3A1 genes, whose expression was significantly correlated with the expression of GDF15 in the GSE126848 dataset (Figure 4C,D).

Interestingly, as shown in Figure S1A–C, the expression pattern of the GDF15, COL1A1, and COL3A1 genes was also confirmed in a previously published mouse model of MASH, in which the GDF15, COL1A1, and COL3A1 transcript levels were all significantly upregulated in the liver of mice with WD-induced MASH compared to that of ND control mice.

**Figure 5:**
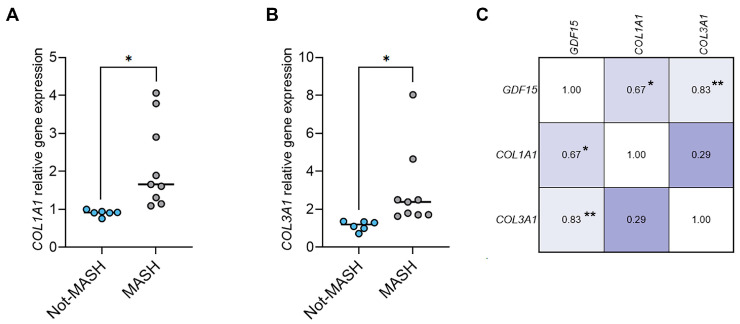
Hepatic gene expression levels of COL1A1 and COL3A1 and correlation with GDF15 gene expression in a subgroup of children with MASLD. The graphs show the relative hepatic gene expression of (**A**) COL1A1 and (**B**) COL3A1 in 9 children with a MASH diagnosis (MASH) and 6 children without MASH (Not-MASH) analyzed by qRT-PCR and normalized to the levels of the β-actin transcript. Unpaired *t*-test with Welch’s correction, * *p* < 0.05. (**C**) Heatmap of the correlations between GDF15, COL1A1, and COL3A1 fold inductions in a subgroup of children with MASH (n = 9), * *p* < 0.05; ** *p* < 0.01.

Finally, we found that also in our subset of 15 patients with MASLD, the hepatic expression of the COL1A1 and COL3A1 transcripts was increased in the liver of children with MASH compared to the liver of those without MASH and that the increased expression of the GDF15 gene was positively correlated with that of the two profibrogenic genes (Figure 5A–C).

## 3. Discussion

In this study, we investigated for the first time the alterations in the plasma and liver levels of GDF15 in children affected by MASLD. Our findings primarily demonstrated that the circulating levels of GDF15 are higher in children with biopsy-proven MASLD compared to controls and increase with the progression of disease damage (i.e., MASH and fibrosis). In addition, we found that hepatic GDF15 gene expression correlated with collagen gene expression in patients and mice with MASH.

To date, identifying biomarkers or panels of biomarkers to detect patients at risk of MASH and severe fibrosis remains an unresolved challenge in adult and pediatric patients [13]. However, in recent years, omics approaches have contributed to the discovery of many molecules that may be able to identify different stages of MASLD, such as GDF15 [14]. Evidence from animal models and humans has implicated GFD15 as an evolutionarily conserved factor in the injury response, which is involved in controlling several processes, including appetite, metabolism, cell and tissue survival, and the immune response [15]. Increased plasma levels of GDF15 have been frequently observed in response to tissue damage and inflammation in both humans and mice [16]. Therefore, it is not surprising that conditions such as obesity and metabolic syndrome, now incorporated in the MASLD diagnostic flowchart, have been associated with significantly increased GDF15 plasma levels.

Over time, studies of individuals diagnosed with MASLD have shown evidence of an association between GDF15 serum levels and the presence and severity of MASLD, also providing an optimal threshold of circulating GDF15 for predicting advanced fibrosis [10,17,18].

Koo et al. [10] reported that higher levels of GDF15 were mainly found in patients with stage 3 or greater fibrosis, as well as in those with the highest liver stiffness values, suggesting that GDF15 may serve as a biomarker for advanced fibrosis in adults with MASLD. Furthermore, a transcriptome analysis of liver samples from adults with biopsy-proven MASLD revealed a strong positive association between hepatic and circulating GDF15 expression levels and the histological signs of advanced liver injury [14]. These results were confirmed by a multicenter study conducted on 374 adult patients with MASLD and 81 age-matched controls [19]. Altogether, these studies corroborated the potential use of GDF15 for the non-invasive identification of the progressive stages of MASLD.

To our knowledge, the present study is the first to explore the significance of GDF15 circulating levels in children with biopsy-diagnosed MASLD. Our study demonstrated that the circulating GDF15 levels were significantly increased in children with MASLD compared to control subjects, and this rise was significantly associated with the severity of the histological pattern of MASLD. Indeed, among the 158 enrolled pediatric patients with MASLD, GDF15 plasma levels were higher in the subgroup with MASH than in children without the advanced form of the disease. In particular, GDF15 expression exhibited a positive correlation with fibrosis. Thus, compared with the study by Galuppo et al. [11], which reported the association of plasma GDF15 with the degree of intrahepatic fat content, here we instead documented the correlation of GDF15 with the histological features that characterize pediatric MASLD. Furthermore, our multivariate regression analysis, adjusted for BMI, body weight, and sex, confirmed that the plasma GDF15 levels were strongly associated with liver fibrosis. This finding is consistent with several studies conducted in adults undergoing liver biopsy or elastography and further strengthens the evidence of an association between circulating GDF-15 and liver fibrosis [9,10,17,18,19]. Indeed, a recent cross-sectional study on 98 adult patients affected by metabolic disorders reported a positive association between GDF15 circulating levels and serum markers of liver injury (AST, ALT, and GGT) and inflammation (i.e., acute-phase proteins) [9]. The study also found a significant association between serum GDF15 levels and hepatic steatosis and fibrosis, measured by the fatty liver index and the Fib-4 index [9]. The authors also highlighted a link between increased GDF15 serum levels and dyslipidemia patterns, thus corroborating our findings regarding the association with triglycerides. Moreover, a very recent study on a large cohort of adult patients with MASLD confirmed that GDF15 circulating levels were significantly increased in patients with moderate to advanced fibrosis (F2–F4). In addition, we found that the GDF15 levels were significantly correlated with the insulin levels, as well as with HOMA-IR values, which are indicators of MASLD in children. Therefore, according to previous studies, our data in children reinforce the involvement of GDF15 as a biomarker for hepato–metabolic damage occurring in MASLD [9,10,17,18,19].

From our results, it also emerged, contrarily to Werge et al. [19], that children with MASH and a murine model of diet-induced MASH displayed a significant increase in hepatic GDF15 gene expression, which was consistent with that observed for the key profibrogenic markers COL1A1 and COL3A1. The latter evidence corroborates the association between soluble and hepatic GDF15 and progression of liver damage. Consistently with our findings, Kim et al. [20] also observed a positive correlation between hepatic GDF15 expression and the presence of MASH in both a mouse model and adult patients with MASH. Interestingly, Patel et al. [21] investigated the crucial aspect of the real hepatic contribution to the elevation in the circulating GDF15 levels in mice with hepatocyte-specific knockdown of GDF15. The authors demonstrated that circulating GDF15 was a reliable predictor of liver damage in high-fat-diet-treated mice, and the liver cells were the primary source of this biomarker’s production.

Concerning the potential involvement of GDF15 in the induction of profibrotic signaling pathways, Koo et al. suggested that GDF15 may act directly on hepatic stellate cells. Indeed, treating LX-2 cells with the GDF15 protein increased the phosphorylation of SMAD2 transcription factors, thus inducing the expression of fibrosis markers such as α-SMA and collagen I [10]. Another hypothesis on the enhancement of GDF15 levels could be linked to the disruption of the intestinal barrier and lipopolysaccharide (LPS) translocation and consequent systemic inflammation in MASLD [22], in line with experimental evidence of LPS-dependent elevation in plasmatic GDF15 levels [23].

Overall, the literature evidence and our findings strongly suggest that GDF15 could be a sensitive and specific biomarker for the non-invasive monitoring of fibrosis progression and patient’s response to treatments [24]. However, whether the pathophysiological modulation of GDF15 expression is a causative or a compensatory effect remains to be determined. Although experimental studies and discontinued trials have demonstrated that treatment with GDF15 is effective in reducing body weight and food intake in obese rodents and primates [25], the role of GDF15 as a therapeutic or target molecule warrants further investigation.

This study has some limitations that should be considered. Firstly, the limited number of controls, due to ethical issues, could influence the strength of our results. Moreover, our investigation was conducted on a small cohort of patients; though this cohort was well characterized, our results could benefit from a validation in a larger independent cohort of children with MASLD. In future research, it would be beneficial to assess the reliability of GDF15 as a non-invasive biomarker of fibrosis in children, comparing its performance with that of liver stiffness or other non-invasive scores, as recently highlighted by Kokkorakis and collaborators [24].

## 4. Materials and Methods

### 4.1. Study Population

We performed a retrospective observational study including 158 enrolled Caucasian children and adolescents (94 males, 64 females) evaluated at the Hepatology and Liver Transplant Unit of the “Bambino Gesù” Children’s Hospital in Rome from March 2019 to March 2022. The diagnosis of MASLD was established by the presence of hepatic steatosis by ultrasound and liver biopsy, alone or in association with at least one of the metabolic risk factors defined by the European Society for Paediatric Gastroenterology Hepatology and the European Association for the Study of the Liver [26]. Patients with a dual cause of liver steatosis (e.g., Wilson’s disease, autoimmune hepatitis, viral hepatitis, endocrinological, genetic, and metabolic diseases, and alcohol and/or drug use) were excluded. The study also included 24 age- and sex-matched healthy control subjects without liver steatosis or other liver diseases, selected from previously obese children who had achieved MASLD resolution during follow-up.

### 4.2. Anthropometric Measurements

Body weight, height, and waist circumference (WC) were measured at the time of diagnosis, with the patient wearing underwear according to a standard protocol. The body mass index (BMI) was calculated using the formula weight (kg)/height (m^2^).

### 4.3. Biochemical Measurements

By using aseptic procedures, blood samples were drawn for measuring ALT, aspartate aminotransferase (AST), gamma-glutamyl transferase (GGT), total cholesterol (TC), high-density lipoprotein cholesterol (HDL-C), low-density lipoprotein cholesterol (LDL-C), uric acid, and fasting glucose and insulin, by using standard laboratory procedures at the Central Laboratory of the “Bambino Gesù” Children’s Hospital. Insulin resistance was assessed using the homeostasis model assessment for insulin resistance (HOMA-IR), with a value greater than 2.5 considered indicative of insulin resistance [27].

### 4.4. Histological Features

Percutaneous liver biopsies were performed using an 18-gauge automatic biopsy needle, general anesthesia, and guided ultrasound.

Each biopsy was evaluated centrally and independently read by two expert liver pathologists to assess NAS (NAFLD activity score) and fibrosis stage (according to NASH Clinical Research Network CRN criteria). Hepatic fibrosis was quantified using a five-point grading: 0 = no fibrosis; 1 = perisinusoidal or periportal fibrosis; 2 = perisinusoidal/periportal portal fibrosis; 3 = fibrous bridge; 4 = cirrhosis [28].

The presence of MASLD and MASH was defined according to the algorithm recently proposed by the Delphi consensus [29]. MASH was defined as NAS ≥ 4, according to the NASH system. Subjects at risk of MASH (i.e., advanced fibrosis or fibrotic MASH) were defined by having NAS ≥ 5 or NAS ≥ 4 and fibrosis F > 1 and compared to a subgroup of Not-MASH subjects that included patients who had a NAS score < 4 and/or fibrosis (F) 0 or 1.

### 4.5. GDF15 Quantification in Human Plasma

After centrifuging the blood at 3000 rpm for 15–20 min, the plasma was stored in tubes at −80 °C and then used to perform an enzyme-linked immunosorbent assay (ELISA). Quantification of GDF15 in plasma samples was conducted by using Human Quantikine ELISA kits (R & D Systems Inc., Minneapolis, MN, USA) according to the manufacturer’s instructions. The assay provides a detection range of 23.4–1500 pg/mL with a threshold of 4.39 pg/mL. Each sample was evaluated in triplicate.

### 4.6. Gene Dataset Analysis

The RNA-seq data were normalized and analyzed using R and DESeq2 (version 4.3.0) [30]. Gene enrichment analysis was performed using Reactome’s pathway analysis tools (https://reactome.org, accessed on 3 July 2024), as previously described [31].

### 4.7. Animal Model

Mouse liver samples were obtained from a previously reported model of diet-induced MASH [32]. Briefly, 2-month-old C57BL/6J mice were randomly divided into two different groups (6 animals per group) and fed for 16 weeks with two types of iso-caloric diets: (i) a normal diet (ND) composed of 18%/w proteins, 44.2%/w carbohydrates, and 6%/w fats (Teklad Global 2018, Harlan Laboratories, Indianapolis, IN, USA); (ii) a Western diet (WD) characterized by 17.3%/w proteins, 48.5%/w carbohydrates, and 21.2%/w fats (TD.88137, Harlan Laboratories Indianapolis, IN, USA). At the end of the treatment, liver tissues were collected and stored at −80 °C for further analysis.

### 4.8. Gene Expression in Human and Mouse Liver Samples

Thirty milligrams of frozen hepatic human and murine tissues were disrupted and homogenized for total RNA extraction using the AllPrep DNA/RNA/Protein Mini Kit (QIAGEN, Hilden, Germany) according to the manufacturer’s protocol. The concentration of the RNA samples was determined by NanoDrop™ 2000/2000 c Spectrophotometers (Thermo Fisher Scientific, Waltham, MA, USA), and the samples were stored at −80 °C until use. Two micrograms of RNA were used to synthesize RNA first-strand complementary DNA (cDNA) by using the SuperScript VILO cDNA Synthesis kit (Thermo Fisher Scientific, Waltham, MA, USA). The cDNAs were measured by quantitative real-time PCR (qRT-PCR) using the 7 Pro RT-PCR System instrument (Thermo Fisher Scientific, Waltham, MA, USA) and SYBR Green Universal Mastermix (Thermo Fisher Scientific, Waltham, MA, USA). The primers were designed using Primer Express software (version 3.0.1) (Thermo Fisher Scientific, Waltham, MA, USA) and purchased from Sigma-Aldrich (St. Louis, MO, USA). The sequences of the designed primers are presented in Table S1. The final data are expressed as foldchange and were normalized by the level of the housekeeping gene β-actin (ACTB) using the following equation:

Fold Change = 2–Δ(ΔCt)

where ΔCt = Ct (target) – Ct (ACTB) and Δ(ΔCt) = ΔCt (treated) – ΔCt (untreated)

### 4.9. Sample Size Calculation

The sample size was calculated to compare the plasma levels of the growth differentiation factor 15 (GDF15) between MASLD and Not-MASH patients, assuming a clinically relevant difference of 50 pg/mL between the two subgroups, with a common standard deviation of 100 pg/mL, based on previously published data [9]. Using a two-sided significance level (α) of 0.05 and a statistical power of 85%, the estimated minimum sample size was calculated based on a two-sample Z-test for the difference between independent means. The estimated minimum sample size was 144 subjects; however, the number of patients was subsequently increased to 158, considering a 10% dropout or missing data rate.

### 4.10. Statistical Analysis

Data are expressed as mean and standard deviation (SD), as medians and interquartile ranges (from the 25th to the 75th percentile), or as frequencies. Differences in clinical variables were assessed using one-way ANOVA for normally distributed continuous variables and the Mann–Whitney U test for non-normally distributed continuous variables.

Pearson and Spearman correlation coefficients were calculated to examine the linear relationships between plasma levels of GDF15 and liver fibrosis values, as well as metabolic parameters. Subsequently, a multivariate linear regression modelling analysis was used to verify the independence of the associations between GDF15 and metabolic and histological parameters after adjusting for potential confounding factors, such as age, gender, and BMI. A *p* < 0.05 was considered statistically significant. Statistical analyses were performed using Medcalc software, version 20.014 (MedCalc Software Ltd., Ostend, Belgium), and GraphPad Prism 10.1.2 (GraphPad Software, Boston, MA, USA).

### 4.11. GenAI

In this manuscript, Google Gemini AI (https://gemini.google.com, accessed on 26 June 2025) was used to perform portions of the graphical abstract.

## 5. Conclusions

In conclusion, our study provides new evidence on the pathophysiologic role of circulating and hepatic GDF15 in pediatric MASLD. In particular, here, we reinforced the role of GDF15 as a possible connector between metabolic and liver derangement occurring in MASLD. Moreover, we highlighted the association between hepatic GDF15 levels and MASH, as well as markers of liver fibrosis, suggesting a potential causative role of GDF15 in fibrosis progression, which warrants further investigation.

## Figures and Tables

**Table 1:** Correlation of GDF15 plasma levels with metabolic, anthropometrical, and biochemical parameters in the pediatric MASH group.

Parameter	Pearson *r*	95% Confidence Interval	*p* Value
Weight	0.09	−0.1053 to 0.2881	0.351
Height	0.04	−0.1606 to 0.2357	0.702
BMI	0.14	−0.0596 to 0.3297	0.168
WC	0.18	−0.0164 to 0.3695	0.718
TC	0.17	−0.0319 to 0.3542	0.099
HDL-C	0.05	−0.0148 to 0.2479	0.610
LDL-C	0.06	−0.1439 to 0.2518	0.583
Triglycerides	0.22	0.02343 to 0.4016	0.003
ALT	0.18	−0.0201 to 0.3645	0.077
AST	0.06	−0.1370 to 0.2583	0.537
GGT	0.19	−0.0051 to 0.3774	0.056
Fasting glucose	0.07	−0.1364 to 0.2649	0.519
Fasting insulin	0.04	−0.1693 to 0.2537	0.686
HOMA-IR	0.13	−0.0675 to 0.3225	0.192
Steatosis	0.06	−0.1351 to 0.2602	0.524
Ballooning	−0.01	−0.2062 to 0.1906	0.936
Lobular inflammation	0.11	−0.0998 to 0.2932	0.324
NAS	0.07	−0.1291 to 0.2658	0.846
Fibrosis	0.49	0.3209 to 0.3626	0.0001

BMI: body mass index; WC: waist circumference; TC: total cholesterol; HDL-C: high-density lipoprotein cholesterol; LDL-C: low-density lipoprotein cholesterol; ALT: alanine aminotransferase; AST: aspartate aminotransferase; GGT: gamma-glutamyl transferase; HOMA-IR: homeostasis model assessment of insulin resistance; NAS: NAFLD activity score. Data are expressed as median (P25–P75 range) with the Mann–Whitney U test.

## Data Availability

The data that support the findings of this study are available from the corresponding author upon reasonable request.

## Supplementary Materials

The following supporting information can be downloaded at: https://www.mdpi.com/article/10.3390/ijms26136486/s1.

## Abbreviations

The following abbreviations are used in this manuscript:

ALT	Alanine aminotransferase
AST	Aspartate aminotransferase
BMI	Body mass index
COL	Collagen
GDF15	Growth differentiation factor 15
GGT	Gamma-glutamyl transferase
HDL-C	High-density lipoprotein cholesterol
HOMA-IR	Homeostasis model assessment for insulin resistance
LPS	Lipopolysaccharides
LDL-C	Low-density lipoprotein cholesterol
MASH	Metabolic dysfunction-associated steatohepatitis
MASLD	Metabolic dysfunction-associated steatotic liver disease
ND	Normal diet
TC	Total cholesterol
WC	Waist circumference
WD	Western diet
