# Supplementary material for: Levels of Growth Differentiation Factor 15 Correlated with Metabolic Dysfunction-Associated Steatotic Liver Disease in Children
Source: Int J Mol Sci. 2025 Jul 5;26(13):6486. doi: 10.3390/ijms26136486 (PMC12249647; doi:10.3390/ijms26136486)

SUPPLEMENTARY DATA

Levels of growth differentiation factor 15 correlated with metabolic dysfunction-associated steatotic liver disease in children

Antonella Mosca[1], Maria Rita Braghini[2], Giulia Andolina[2], Cristiano De Stefanis[3], Lucia Cesarini[2], Anna Pastore[2], Donatella Comparcola[1], Lidia Monti[4], Paola Francalanci[5], Clara Balsano[6,7], Andrea Pietrobattista[1], Anna Alisi[2§], Nadia Panera[2]

[1]*Hepatology and Liver Transplant Unit, Bambino Gesù Children's Hospital, IRCCS, Rome, Italy*

[2]*Research Unit of Genetics of Complex Phenotypes, Bambino Gesù Children's Hospital, IRCCS, Rome, Italy.*

[3]*Core Facilities, Bambino Gesù Children's Hospital, IRCCS, Rome, Italy.*

[4]*Operation Unit Radiology, Bambino Gesù Children's Hospital, IRCCS, Rome, Italy.*

[5]*Molecular Pathology Research Unit, Bambino Gesù Children's Hospital, IRCCS, Rome, Italy.*

[6]*Department of Life, Health and Environmental Sciences-MESVA, School of Emergency and Urgency Medicine, Geriatric Unit, University of L'Aquila, L'Aquila, Italy.*

[7]*Francesco Balsano Foundation, Via Giovanni Battista Martini 6, Rome, 00198, Italy*

[§]**Corresponding author:**

Anna Alisi, PhD, Research Unit of Genetics of Complex Phenotypes, Bambino Gesù Children's Hospital, IRCCS, Rome, Italy. anna.alisi@opbg.net.

Checklist

Table S1, page 3

Table S2, page 4

Table S3, page 5

Table S4 page 6

Table S5, page 7

Table S6, page 8

Figure S1, page 9

Table S1 Primers used for human and mouse reverse qRT-PCR.

Primers	Direction	Human	Mouse
GDF15	REV	CAACCAGAGCTGGGAAGATTCG	AGCCGAGAGGACTCGAACTCAG
	FDW	CCCGAGAGATACGCAGGTGCA	GGTTGACGCGGAGTAGCAGCT
COL1A1	REV	GCCAAGACGAAGACATCCCACC	CCTCAGGGTATTGCTGGACAAC
	FDW	TCCCTTGGGTCCCTCGACG	CAGAAGGACCTTGTTTGCCAGG
COL3A1	REV	CTGGTGCTAAGGGTGAAGTTG	GACCAAAAGGTGATGCTGGACAG
	FDW	CCAGCAGGACCCTTTTCTC	CAAGACCTCGTGCTCCAGTTAG
ACTB	REV	CACCATTGGCAATGAGCGGTTC	CATTGCTGACAGGATGCAGAAGG
	FDW	AGGTCTTTGCGGATGTCCACGT	TGCTGGAAGGTGGACAGTGAGG

Table S2 Clinical characteristics of the study population.

Variables	Controls (N = 24)	MASLD (N = 158)	p value
Age, years	12 (9-14.50)	13.00 (11.00-16.00)	0.075
Sex, F/M	11/13	64/94	-
Height, cm	140 (126.50-150.50)	158.00 (148.5-168.00)	**0.003**
Weight, kg	39.4 (26.85-54.28)	72.5 (57.38-89.05)	**<0.0001**
BMI, kg/sqm	19.1 (17.09-19.24)	28.05 (25.05-34.15)	**<0.0001**
ALT, UI/L	23 (18.50-26.50)	27.00 (24.00-37.00)	**<0.0012**
AST, UI/L	22 (18.75-25.25)	29.00 (21.75-37.25)	**0.005**
Triglycerides, mg/dL	75 (60.50-88.25)	93.00 (62.00-138.50)	**0.0287**
TC, mg/dL	144.50 (108.30-161.50)	151.50 (133.00-168.00)	0.0524
HDL-C, mg/dL	42 (39.00-49.75)	45.00 (39.00-53.00)	0.428
LDL-C, mg/dL	82.00 (65.75-89.50)	94.00 (73.00-106.50)	**0.012**
Fasting glucose, mg/dL	78.50 (77.25-80.00)	83.00 (77.00-89.00)	**0.172**

MASLD: metabolic dysfunction-associated steatotic liver disease: F: female; M: male; BMI: body mass index; ALT: alanine aminotransferase; AST: aspartate aminotransferase; TC: total cholesterol; HDL-C: high-density lipoprotein-cholesterol; LDL-C: low-density lipoprotein-cholesterol. Data are expressed as median (P25-P75 range) with the Mann-Whitney U test.

Table S3 Correlation of GDF15 plasma levels with metabolic, anthropometric, and biochemical parameters in pediatric MASLD group.

Parameters	Pearson r	95% confidence interval	*p* value
Age	0.07	-0.085 to 0.2253	0.370
Weight	0.13	-0.0189 to 0.2876	0.084
Height	0.02	-0.1317 to 0.1804	0.750
BMI	0.21	-0.0570 to 0.3557	**0.007**
WC	0.16	0.0029 to 0.3084	0.056
TC	0.15	-0.0004 to 0.3000	0.057
HDL-C	0.06	-0.0891 to 0.2217	0.396
LDL-C	0.09	-0.0597 to 0.2497	0.224
Triglycerides	0.19	-0.0425 to 0.3429	**0.013**
ALT	0.16	0.0117 to 0.3154	**0.035**
AST	0.05	-0.1067 to 0.2049	0.530
GGT	0.16	0.0022 to 0.3069	**0.047**
Fasting glucose	-0.04	-0.1986 to 0.1162	0.601
Fasting insulin	0.23	0.0760 to 0.3834	**0.004**
HOMA-IR	0.17	0.0194 to 0.3224	**0.027**
Fibrosis	0.43	0.296 to 0.5512	**<0.001**
Steatosis	0.24	0.092 to 0.3864	**0.002**
Inflammation	0.27	0.1286 to 0.4171	**<0.001**
Ballooning	0.13	-0.0197 to 0.2868	0.086

BMI: body mass index; WC: waist circumference; TC: Total cholesterol; HDL-C high-density lipoprotein-cholesterol; LDL-C: low-density lipoprotein-cholesterol; ALT, alanine aminotransferase; AST: aspartate aminotransferase; GGT: gamma-glutamyl transferase; HOMA-IR: homeostasis model assessment of insulin resistance.

Table S4. Histological features of children with MASLD.

Histological features	N	%
Steatosis		
1	71	44.9
2	71	44.9
3	16	10.1
Lobular inflammation		
0	16	10.1
1	92	58.2
2	50	31.7
Ballooning		
0	24	15.2
1	86	54.4
2	48	30.4
Fibrosis		
0	30	19
1	51	32.2
2	66	41.8
3	11	7.0
NAS		
0	0	0
1	8	5.1
2	23	14.5
3	24	15.2
4	5	3.2
5	81	51.1
6	17	10.8
7	0	-

NAS: NAFLD Activity Score

Table S5 Clinical and biochemical characteristics of children with MASLD, stratified by presence or absence of MASH.

**Variables**	**Not-MASH (N = 60)**	**MASH (N = 98)**	***p* value**
Age, years	13.4 (11.30, 15.70)	13.00 (11.00-16.00)	0.3034
Sex, F/M	26/34	38/60	-
Height, cm	158.00 (154.00-166.50)	157.00 (144.0-169)	0.9871
Weight, kg	75.2 (63.6.00-86.00)	71.6 (55.00-98)	0.7792
BMI, kg/sqm	28.00 (25.66-32.99)	28.15 (24.23-35.69)	0.9234
WC, cm	94 (84.5.00-9.50)	90.00 (81-103.00)	0.6140
ALT, UI/L	26.00 (21.00-28.50)	32 (26-40.00)	**<0.0001**
AST, UI/L	25.00 (20.00-35.50)	31.00 (24-39.00)	**0.0041**
GGT, UI/L	16.5.0 (11.00-21.00)	17.00 (13-24)	0.8879
Triglycerides, mg/dL	86.50 (57.00-109.5)	107.3 (63.00-149.00)	0.0542
TC, mg/dL	150.50 (132.00-171.00)	154.00 (136-168)	0.8200
HDL-C, mg/dL	46.5 (40.00 -60.00)	44.50 (38.00-50.00)	0.2154
LDL-C, mg/dL	82.00 (67.00-101.00)	95.50 (75.00-110.00)	**0.0467**
Fasting glucose, mg/dL	80.00 (74.00-89.00)	84 (78-90.00)	0.8378
Fasting insulin, UI/mL	14.33 (10.45-18.00)	18.35 (10.80-28.20)	**0.0087**
HOMA-IR	2.79 (2.05-03.75)	3.67 (2.38-05.39)	**0.024**

F: female; M: male; BMI: body mass index; WC: waist circumference, ALT: alanine aminotransferase, AST: aspartate aminotransferase; GGT: gamma-glutamyl transferase, TC: total cholesterol, HDL-C: high-density lipoprotein-cholesterol, LDL-C: low-density lipoprotein-cholesterol, HOMA-IR: homeostasis model assessment of insulin resistance. Data are expressed as median (P25-P75 range) with Mann-Whitney U test.

Table S6 List of genes positively correlated with the *GDF15* gene in a hepatic dataset from adults with MASLD.

This supplementary Table is provided as an Excel file.

Figure S1 Gene expression levels of *GDF15*, *COL1A1*, and *COL3A1* in a murine model of MASH. The histograms report the murine relative hepatic gene expression of (A) *Gdf15*, (B) *Col1a1*, and (C) *Col3a1* analyzed by qRT-PCR and normalized to β-Actin transcript. Data are expressed as the mean ± SD of at least n = 5 independent murine liver samples per group. ND= normal diet; WD=western diet. Unpaired t-test with Welch's correction, *p<0.05; *** p<0.001.

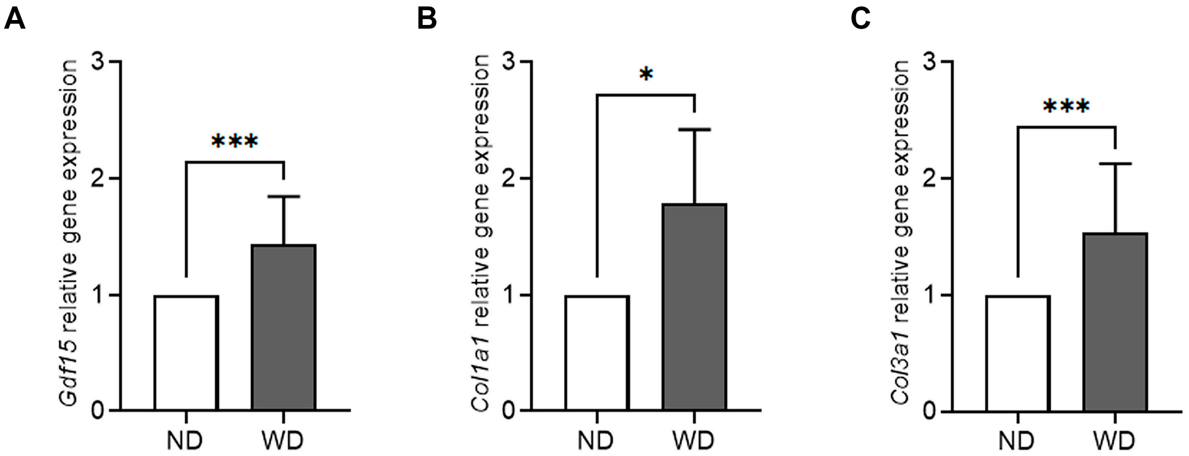

Supplement: Supplementary file 1 [file ijms-26-06486-s001.zip › ijms-3713924-supplementary.pdf]
